# OpenAI’s Sora: it’s time to update the dissemination methods of medical findings

**DOI:** 10.1097/JS9.0000000000001920

**Published:** 2024-07-02

**Authors:** Yuanjun Lyu, Zaijie Sun, Qiang Guo, Cheng Li, Haiyang Wu

**Affiliations:** a Department of Geriatric Respiratory and Sleep, The First Affiliated Hospital of Zhengzhou University; b Department of Orthopaedics, The First Affiliated Hospital of Zhengzhou University, Zhengzhou; c Department of Spine and Joint Surgery, Tianjin Baodi Hospital, Baodi Clinical College of Tianjin Medical University, Tianjin; d Department of Spine Surgery, Wangjing Hospital, China Academy of Chinese Medical Sciences, Beijing; e Department of Orthopaedic Surgery, Xiangyang Central Hospital, Affiliated Hospital of Hubei University of Arts and Science, Xiangyang, People’s Republic of China; f Center for Musculoskeletal Surgery (CMSC), Charité-Universitätsmedizin Berlin, Corporate Member of Freie Universität Berlin, Humboldt University of Berlin, Berlin Institute of Health, Berlin, Germany

The fervor surrounding the utilization of the large language model, ChatGPT, remains unabated^1-2^. On 16 February 2024, OpenAI introduced Sora, a text-to-video model heralding a significant advancement in artificial intelligence (AI) technology. Unlike its predecessors, Sora possesses the capability to fabricate sleek, high-resolution videos spanning a duration of 60 s, crafted solely from prompt words. Its repertoire includes an array of vibrant hues and intricate camera maneuvers, symbolizing a paradigm shift in generative AI from textual and pictorial realms to the domain of video production^3^.

In comparison to existing video generation models like DALL-E, GENMO, and PIKA, Sora has attained unprecedented milestones in duration, video quality, and adherence to physical laws. While the prevailing standard for video generation stands at a mere ‘3-4s,’ Sora could produce videos up to 1 min in length, all while meticulously adhering to user prompts and maintaining exceptional video quality. Furthermore, Sora boasts profound linguistic comprehension, enabling it to construct emotionally resonant characters and intricate scenarios, and possesses the capacity to decipher and engage with real-world contexts. Many scholars view Sora as a watershed moment in artificial intelligence’s capacity to interpret and visualize temporal narratives, underscoring its profound implications^4-5^. Leveraging Sora’s considerable potential in video generation, it is imperative to explore its prospective applications in the medical domain.

Firstly, within the realm of surgical planning, whereas ChatGPT is confined to text-based blueprints, Sora empowers the generation of comprehensive surgical videos, offering visual insights into procedural intricacies. These visual aids not only facilitate preoperative planning and risk assessment but also mitigate surgical hazards, thereby enhancing patient safety.

In medical pedagogy and training, Sora’s ability to generate high-fidelity instructional videos could be invaluable. Different from ChatGPT’s personalized interaction and instant feedback, Sora can effectively convey complex medical information with its vivid visual and multi-sensory experience and is suitable for large-scale medical education and promotion^6^. These videos could elucidate intricate medical procedures and case studies, serving as vital educational resources for aspiring medical professionals. Additionally, medical case analysis videos foster a deeper understanding of diagnostic methodologies and treatment protocols, thereby refining clinical acumen.

In addition, Virtual Reality (VR) is a technology that uses computer systems to create immersive virtual environments, allowing users to experience a simulated world through multiple senses such as sight and sound. The application of VR technology in the field of surgery offers significant advantages^7^. Firstly, through VR, surgeons could access essential patient data, such as ultrasound, mammography, CT, and MRI scans, in real time during surgical procedures. This information, traditionally limited to 2D flat screens, could now be projected directly into the operating room. This eliminates the need for surgeons to frequently check a separate screen, allowing them to focus entirely on the patient. Additionally, VR headsets enable surgeons to instantly access all necessary digital files, images, and data, thereby enhancing the precision and efficiency of surgeries. Taking spine surgery as an example, the portability and image projection capabilities of VR greatly benefit spine surgeons. During procedures, VR technology simplifies the insertion of pedicle screws and eliminates the need for surgeons to divert their gaze to other devices. Real-time surgical guidance through 3D visualization and augmented reality significantly improves the accuracy and efficiency of spine surgeries. High-fidelity surgical videos generated by text-to-video tools, combined with VR technology, allow medical students to practice surgeries in a virtual environment, reducing the reliance on actual human and animal experiments.

In the realm of public health education, videos emerge as potent tools for disseminating medical knowledge. Their visual and auditory appeal renders complex medical concepts more accessible, facilitating enhanced comprehension and retention. Moreover, their versatility enables widespread dissemination across various online platforms (such as YouTube and TikTok), transcending geographic and demographic barriers and reaching diverse audiences, including medical practitioners, patients, and the general populace. For example, in the early stages of the COVID-19 pandemic, the World Health Organization (WHO) produced a series of short videos about protective measures against the coronavirus. These videos were rapidly disseminated through social media platforms, enhancing public awareness and knowledge of protective measures^8^.

More importantly, AI also could play a crucial role in drug discovery and development, as well as clinical trials^9-11^. By combining natural language processing and computer vision technologies, OpenAI’s Sora could support various aspects of drug research. First, drug development generates vast amounts of complex data, such as genomic and protein structure data. Sora is able to transform this complex data into intuitive videos, aiding researchers in better understanding and analyzing the information^12-13^. Additionally, this technology could create virtual laboratories to simulate drug synthesis, screening, and testing processes, reducing the risks and costs associated with actual operations. Furthermore, dynamically displaying molecular interactions helps researchers better understand the relationships between drugs and their targets. Finally, the ultimate goal of drug development is public use. Through engaging videos, Sora could translate complex scientific discoveries into easily understandable content, explaining the progress and significance of drug research to the public and investors.

Lastly, traditional academic discourse predominantly relies on written mediums such as papers and reports. While text effectively communicates intricate academic concepts, its efficacy is marred by reader fatigue. In contrast, video content, with its audio-visual presentation, offers a more engaging and dynamic medium for conveying abstract notions and research findings. Particularly in elucidating complex experimental procedures or research methodologies, videos offer unparalleled clarity, fostering deeper understanding and mastery. As such, the incorporation of video content into academic communication is poised to assume greater prominence. Notably, an increasing number of scholarly journals advocate for the creation of video abstracts for dissemination on public platforms, indicative of the burgeoning role of video content in scholarly discourse. The advent of Sora portends a transformative trajectory for medical video production, promising to catalyze advancements in academic communication and knowledge dissemination.

## Ethical approval

This study does not include any individual-level data and thus does not require any ethical approval.

## Source of funding

This study is supported by China Postdoctoral Science Foundation (2022M720385) and Beijing JST Research Funding (YGQ-202313).

## Author contribution

Y.L.: conceptualization, methodology, data curation, formal analysis, resources, investigation, and writing – original draft; Z.S. and Q.G.: conceptualization, methodology, data curation, formal analysis, resources, and investigation; H.W. and C.L.: methodology, data curation, formal analysis, resources, investigation, and writing – review and editing.

## Conflicts of interest disclosure

The authors declare no conflicts of interest.

## Research registration unique identifying number (UIN)

1. Name of the registry: not applicable.
2. Unique identifying number or registration ID: not applicable.
3. Hyperlink to your specific registration (must be publicly accessible and will be checked): not applicable.

## Guarantor

Haiyang Wu and Cheng Li.

## Data availability statement

The data underlying this article will be shared by the corresponding author on reasonable request.
